# Enhancement of the immunogenicity of a *Mycobacterium tuberculosis* fusion protein using ISCOMATRIX and PLUSCOM nano-adjuvants as prophylactic vaccine after nasal administration in mice

**DOI:** 10.22038/IJBMS.2023.69295.15100

**Published:** 2024

**Authors:** Arshid Yousefi Avarvand, Zahra Meshkat, Farzad Khademi, Ehsan Aryan, Mojtaba Sankian, Mohsen Tafaghodi

**Affiliations:** 1 Department of Laboratory Sciences, School of Allied Medical Sciences, Ahvaz Jundishapur University of Medical Sciences, Ahvaz, Iran; 2 Antimicrobial Resistance Research Center, Mashhad University of Medical Sciences, Mashhad, Iran; 3 Department of Medical Bacteriology and Virology, School of Medicine, Mashhad University of Medical Sciences, Mashhad, Iran; 4 Department of Microbiology, School of Medicine, Ardabil University of Medical Sciences, Ardabil, Iran; 5 Immunobiochemistry laboratory, Immunology Research Center, Bu-Ali Research Institute, Mashhad, Iran; 6 Nanotechnology Research Center, Pharmaceutical Technology Institute, Mashhad University of Medical Sciences, Mashhad Iran

**Keywords:** HspX/EsxS, ISCOMATRIX, MPLA, Mycobacterium, tuberculosis, Nasal administration, PLUSCOM

## Abstract

**Objective(s):**

Tuberculosis (TB), a contagious disease caused by *Mycobacterium tuberculosis* (*M. tuberculosis*), remains a health problem worldwide and this infection has the highest mortality rate among bacterial infections. Current studies suggest that intranasal administration of new TB vaccines could enhance the immunogenicity of *M. tuberculosis* antigens. Hence, we aim to evaluate the protective efficacy and immunogenicity of HspX/EsxS fusion protein of *M. tuberculosis* along with ISCOMATRIX and PLUSCOM nano-adjuvants and MPLA through intranasal administration in a mice model.

**Materials and Methods:**

In the present study, the recombinant fusion protein was expressed in Escherichia coli and purified and used to prepare different nanoparticle formulations in combination with ISCOMATRIX and PLUSCOM nano-adjuvants and MPLA. Mice were intranasally vaccinated with each formulation three times at an interval of 2 weeks. Three weeks after the final vaccination, IFN-γ, IL-4. IL-17, and TGF-β concentrations in the supernatant of cultured splenocytes of vaccinated mice as well as serum titers of IgG1 and IgG2a and sIgA titers in nasal lavage were determined.

**Results:**

According to obtained results, intranasally vaccinated mice with formulations containing ISCOMATRIX and PLUSCOM nano-adjuvants and MPLA could effectively induce IFN-γ and sIgA responses. Moreover, both HspX/EsxS/ISCOMATRIX/MPLA and HspX/EsxS/PLUSCOM/MPLA and their BCG booster formulation could strongly stimulate the immune system and enhance the immunogenicity of *M. tuberculosis* antigens.

**Conclusion:**

The results demonstrate the potential of HspX/EsxS-fused protein in combination with ISCOMATRIX, PLUSCOM, and MPLA after nasal administration in enhancing the immune response against *M. tuberculosis* antigens. Both nanoparticles were good adjuvants in order to promote the immunogenicity of TB-fused antigens. So, nasal immunization with these formulations, could induce immune responses and be considered a new TB vaccine or a BCG booster.

## Introduction

Tuberculosis (TB) is a contagious disease with approximately 1.7 billion latently infected people and over 1.2 million deaths annually. TB is among the 10 causes of death worldwide, according to the latest World Health Organization (WHO) report, which can be controlled using early vaccination as well as rapid detection and treatment with the first- and second anti-TB drugs (1-4). However, the emergence of *Mycobacterium tuberculosis *resistant strains particularly rifampicin-resistant and multidrug-resistant TB (MDR) has led to treatment failures (5). Furthermore, for many years, the existence of some disadvantages in the only licensed* M. tuberculosis* vaccine, BCG (Calmette-Guérin Bacillus), has led to many efforts to assess the other ways of controlling the TB disease (6, 7). The efficacy of the BCG vaccine for pulmonary TB decreases during the lifetime and therefore it is more effective against newborns and children (8, 9). Additionally, BCG is not recommended for patients with immune deficiency and is not able to control latent TB infections which can act as reservoir of active TB infection (3). Therefore, several vaccines are in different steps of clinical or preclinical studies. These vaccines are examined for either pre-exposure prevention which can be administrated before TB infection in newborns and adolescents or post-exposure and therapeutic vaccines which can be administered in adolescents and adults after TB infection to eliminate latent TB. These new types of TB vaccines are considered as either alternatives for the BCG vaccine or as boosters of BCG prime (10-12). Multi-stage subunit vaccines as pre-exposure, post-exposure, and therapeutic vaccines, are promising candidates for boosting BCG-primed immunity or a prime-vaccine alternative for BCG vaccine (10, 13). On the other hand, combining multi-stage subunit vaccines with adjuvants and delivery systems can potentiate the immunogenicity of multi-stage vaccines, protect antigens from enzymatic degradation and *in vivo* elimination, targeted delivery and then efficient uptake of antigens and control of antigens release (14, 15). In a series of the studies, we evaluated the potential of a novel multicomponent subunit vaccine candidate called HspX/EsxS-fused protein, a latent-phase protein (HspX) plus an early-phase protein (EsxS), along with various adjuvants such as DOTAP (1, 2-dioleoyl-3-trimethylammonium propane), MPLA (monophosphoryl lipid A), and DDA (dimethyl dioctadecylammonium bromide), as well as delivery systems such as PLGA (poly (lactide-co-glycolide)) through different administration routes in animal models in order to enhance the immunogenicity of *M. tuberculosis* antigens (16-18). Furthermore, two nano-adjuvants ISCOMATRIX, a negatively charged particle, and PLUSCOM, a positively charged ISCOMATRIX, were also evaluated along with HspX/EsxS-fused protein via subcutaneous administration and the results were promising in animal models (unpublished data). However, as *M. tuberculosis* enters via the respiratory tract, the mucosal administration of these formulations might rapidly induce the innate and adaptive immune responses at the respiratory mucosal surfaces (10, 19, 20). Therefore, we followed two aims; 1) determine the potential of HspX/EsxS-fused protein in combination with ISCOMATRIX, PLUSCOM, and MPLA after nasal administration, and 2) comparison of the current results with our previous results.

## Materials and Methods

Phosphatidylcholine was purchased from Avanti polar lipids (USA). Sucrose was from Merck (Germany). Saponin, BSA, MPLA, DDA, and dimethyl dioctadecylammonium bromide were purchased from Sigma-Aldrich (USA). FBS and PHA were purchased from Thermo Fisher Scientific (USA). Pen-Strep and RPMI were from Biosera (USA). BCA Protein Quantification Kit was from Parstous Biotechnology (Iran). BALB/c mice were provided by Pasteur Institute (Iran). Mouse IFN gamma ELISA Ready-SET-GO kit, Mouse IL-4 ELISA Ready-SET-GO kit, Mouse IL-17A ELISA Ready-SET-Go kit, and Mouse TGFbeta1 ELISA Ready-SET–Go kit were purchased from eBioscience (USA). Goat anti-Mouse IgA Secondary Antibody, HRP conjugate and Goat anti-Mouse IgG1 Secondary Antibody, HRP conjugate were from Invitrogen (USA).

***Preparation of HspX/EsxS protein***

Synthesis of the HspX/EsxS fused protein was performed as described previously. To perform this, the recombinant fusion protein was expressed in *Escherichia coli*, purified on a chromatography column (Parstous Biotechnology, Iran), and then verified by SDS-PAGE and western blot. The protein concentration was also measured by a BCA kit (Parstous Biotechnology, Iran) (16). 

***Preparation of ISCOMATRIX and PLUSCOM nano-adjuvants***

ISCOMATRIX and PLUSCOM nano-adjuvants were prepared by the lipid film hydration method. Briefly, to provide the ISCOMATRIX nano-adjuvant, 200 µl of cholesterol (4 mg/ml) along with 320 µl of phosphatidylcholine (8 mg/ml) (Avanti polar lipids, USA) were dissolved in dichloromethane and then mixed and vacuum dried to eliminate the dichloromethane and establish the lipid film. The PLUSCOM lipid film was also prepared by mixing 200 µl of DDA (4, 8, or 16 mg/ml) and 320 µl of phosphatidylcholine (8 mg/ml) dissolved in dichloromethane. Both ISCOMATRIX and PLUSCOM lipid films were hydrated by 200 mg of sucrose (Merck, Germany), dissolved in distilled water (2 ml) and butanol (2 ml), and then freeze-dried overnight. The freeze-dried powders were combined with an aqueous phase containing saponin (8 mg in 4 ml of PBS (0.01 M), pH 7.4) (Sigma-Aldrich, USA) and then bath sonicated (Kerry, UK) at 37 °C for 10 min. Dynamic light scattering (DLS) (Zetasizer Nano, Malvern, UK) was used to measure the particle size and surface charge of nano-adjuvants (20-23).

***Animals***

Fifty female BALB/c mice, 6 to 8 weeks old, with an approximate weight of 16 to 23 grams in each group were obtained from Bu-Ali Research Institute of Mashhad and were kept in the animal room under 12-hr light/dark cycle, according to the standards of the ethics committee of Mashhad University of Medical Sciences. Mice were divided into 10 groups (5 animals each) for the study of different formulations.

***Prophylactic vaccination of animal model***

The following vaccine formulations were prepared in aseptic conditions for nasal administration of 10 female mice groups including 5 BALB/c mice, 6 to 8 weeks old, in each group: 1) PBS (negative control), 2) BCG (5×10^5^ CFU/mouse), 3) HspX/EsxS, 4) HspX/EsxS/MPLA, 5) HspX/EsxS/ISCOMATRIX, 6) HspX/EsxS/PLUSCOM, 7) HspX/EsxS/ISCOMATRIX/MPLA, 8) HspX/EsxS/PLUSCOM/MPLA, 9) HspX/EsxS/ISCOMATRIX/MPLA as BCG booster, and 10) HspX/EsxS/PLUSCOM /MPLA as BCG booster. Mice were nasally vaccinated with 20 µl of each formulation (10 µg of HspX/EsxS, 15 µg of ISCOMATRIX, 15 µg of PLUSCOM, and 15 µg of MPLA) three times at an interval of 2 weeks. In the case of BCG booster groups (groups 9-10), BCG injection with the relevant formulation was performed on day 0, and relevant formulations were prescribed on days 14 and 28. Groups 7 and 8 did not receive BCG booster.

***Assessment of HspX/EsxS antigen-specific cytokines secreted by spleen cells***

Three weeks after the final vaccination, all mice were sacrificed by cervical dislocation, nasal lavage, blood and spleen of vaccinated mice were used to assay IgA, IgG1, and IgG2a titers as well as interferon-gamma (IFN-γ), interleukin 4 (IL-4), interleukin 17 (IL-17), and transforming growth factor beta (TGF-β) cytokines (18, 24). For cytokine assays, production of IFN-γ, IL-4, IL-17, and TGF-β by splenic lymphocytes (2 × 10^6^ cells/well) of mice which were stimulated with each formulation, were measured in the supernatant of cultured splenocytes according to the enzyme-linked immunosorbent assay (ELISA) kit (eBioscience, USA) (20). 

***HspX/EsxS antigen-specific antibody assay***

Additionally, goat anti-mouse IgA:HRP, IgG1:HRP, and IgG2a:HRP (Invitrogen, USA) were used for measurement of lavage anti-HspX/EsxS IgA titers and serum anti-HspX/EsxS IgG1 and IgG2a titers. In the case of splenic lymphocytes, cell supernatant has been used to measure cytokines (20).

***Statistical analysis***

All statistical analyses were performed using the GraphPad Prism 8.0 software, and all data analysis was performed by one-way ANOVA in combination with Tukey’s multiple comparison tests. Values were expressed as mean ± SD, when *P*-value<0.05, differences were considered statistically significant. Significance was presented as *(*P*<0.05), **(*P*<0.01), ***(*P*<0.001), and ****(*P*<0.0001), and not significant was shown as (ns).

## Results

***Assessment of IFN-γ response***

**Figure 1:**
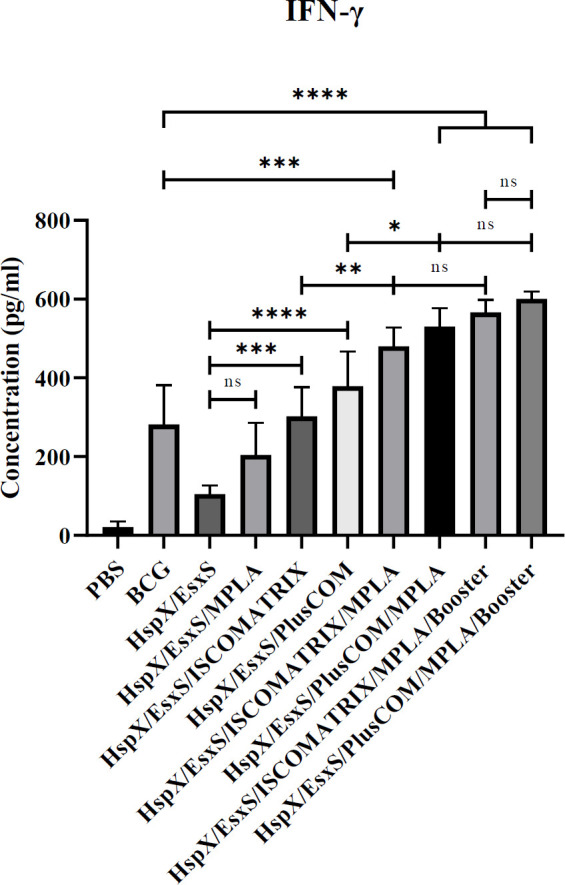
Level of IFN-γ produced in the spleen cells of the mice receiving different formulations

After nasal administration, our results showed that formulations containing nano-adjuvants ISCOMATRIX (ISCOMATRIX/HspX/EsxS) and PLUSCOM (PLUSCOM/HspX/EsxS) were able to boost HspX/EsxS immunogenicity and induced higher levels of IFN-γ response compared to HspX/EsxS alone, (*P*<0.001) and (*P*<0.0001), respectively. Also, the addition of MPLA adjuvant to ISCOMATRIX/HspX/EsxS and PLUSCOM/HspX/EsxS formulations promoted the immune responses. The spleen cells of the mice receiving HspX/EsxS/ISCOMATRIX/MPLA and HspX/EsxS/PLUSCOM/MPLA formulations significantly produced higher levels of IFN-γ than those receiving HspX/EsxS/ISCOMATRIX and HspX/EsxS/PLUSCOM, respectively (*P*<0.01 and *P*<0.05). There was no significant difference between BCG boosters of HspX/EsxS/ISCOMATRIX/MPLA and HspX/EsxS/PLUSCOM/MPLA (*P*>0.05), although, both HspX/EsxS/ISCOMATRIX/MPLA and HspX/EsxS/PLUSCOM/MPLA and their BCG booster formulation were able to induce IFN-γ response significantly higher than the BCG group (*P*<0.001) (Figure 1).

***Assessment of IL-17*** ***response***

**Figure 2:**
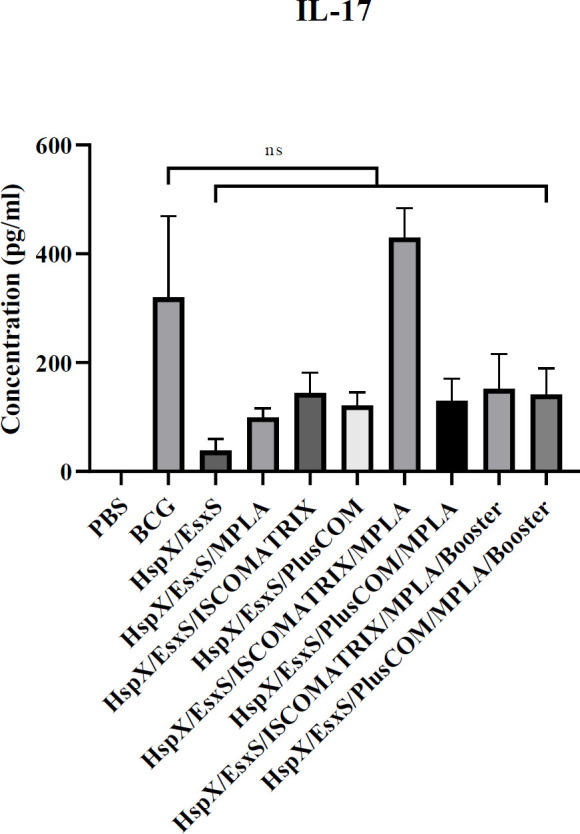
Level of IL-17 produced in the spleen cells of the mice receiving different formulations

Our results show that different formulations did not induce IL-17 response significantly in the stimulated splenic lymphocytes of mice compared to the BCG group (*P*>0.05) (Figure 2).

***Assessment of IL-4*** ***response***

**Figure 3:**
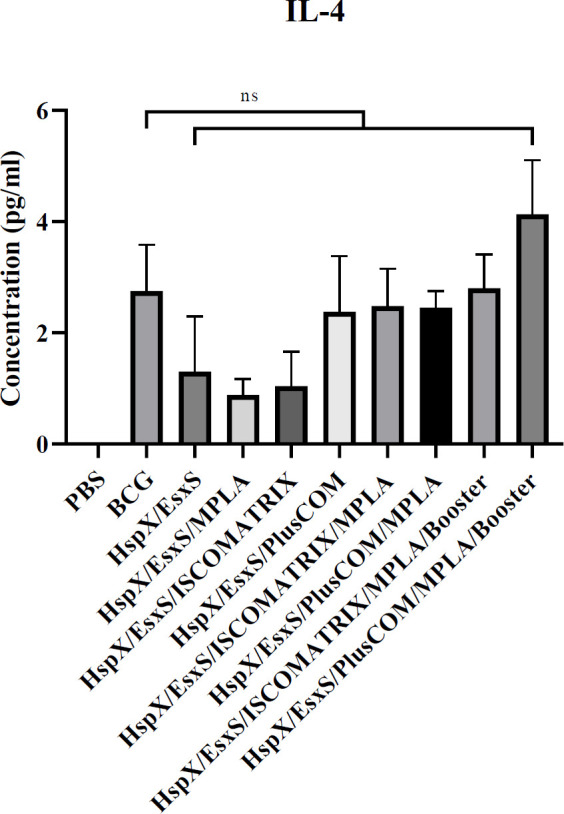
Level of IL-4 produced in the spleen cells of the mice receiving different formulations

According to obtained results, the level of IL-4 secretion in vaccinated mice with BCG booster of HspX/EsxS/PLUSCOM/MPLA formulation was higher than HspX/EsxS and BCG vaccine (*P*>0.05). However, no formulations were able to induce IL-4 response significantly higher than the BCG group (*P*>0.05) (Figure 3).

***Assessment of TGF-β*** ***response***

**Figure 4:**
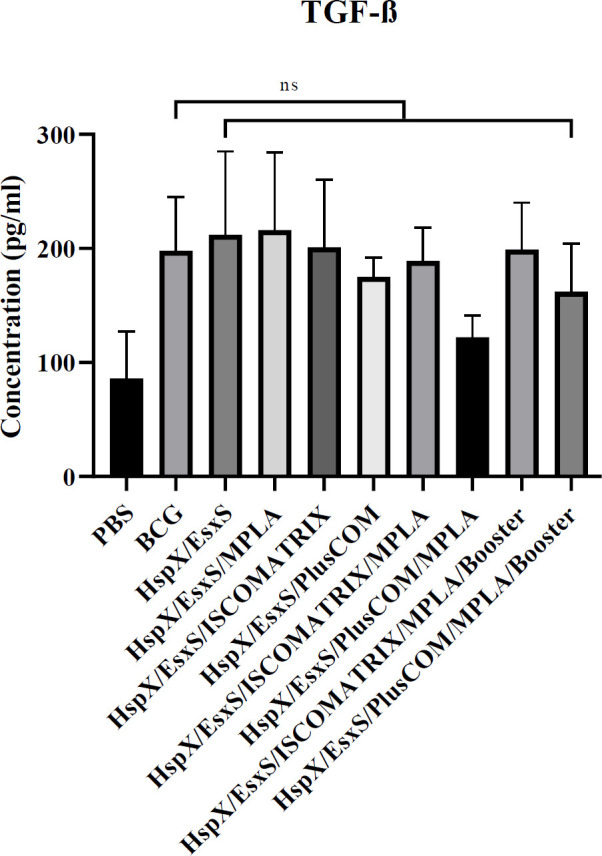
Level of TGF-β produced in the spleen cells of the mice receiving different formulations

Similar to IL-4 and IL-17, there was no significant difference between different formulations and the BCG group in the induction of TGF-β response (*P*>0.05) (Figure 4).

***Assessment of IgG2a antibody response***

**Figure 5.**
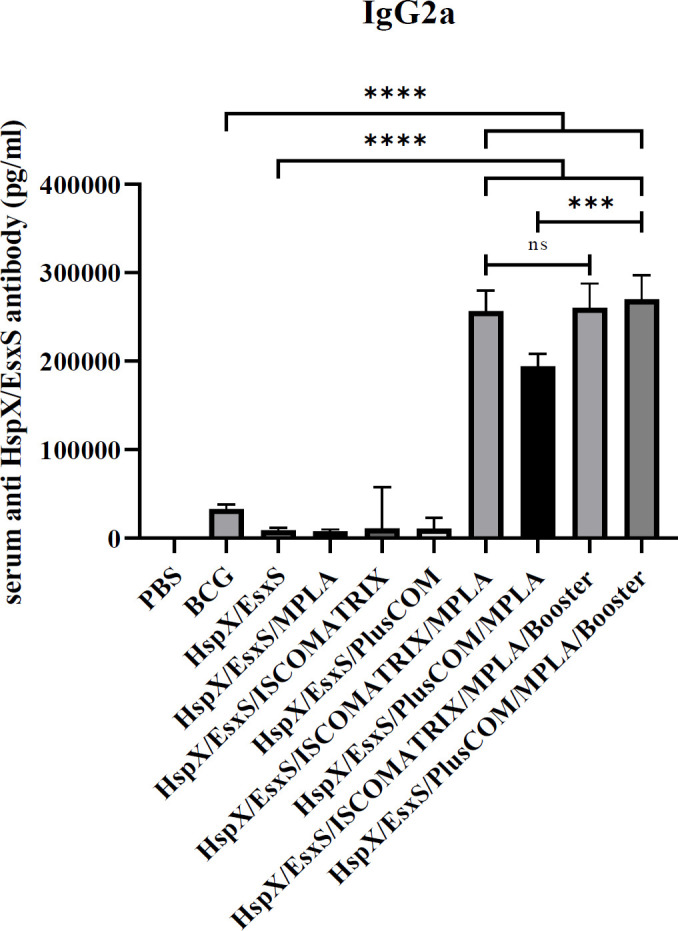
Level of IgG2a produced in the serum of mice receiving different formulations

On the 50^th^ day, three weeks after the last vaccination, the levels of serum anti-HspX/EsxS IgG2a titers in mice vaccinated with HspX/EsxS/ISCOMATRIX/MPLA, HspX/EsxS/PLUSCOM/MPLA, and their BCG booster formulations were significantly higher than HspX/EsxS and BCG vaccines (*P*<0.0001). Additionally, HspX/EsxS/PlusCOM/MPLA/Booster was able to significantly increase IgG2a responses higher than HspX/EsxS/PlusCOM/MPLA (*P*<0.001) (Figure 5).

***Assessment of IgG1 antibody response***

**Figure 6:**
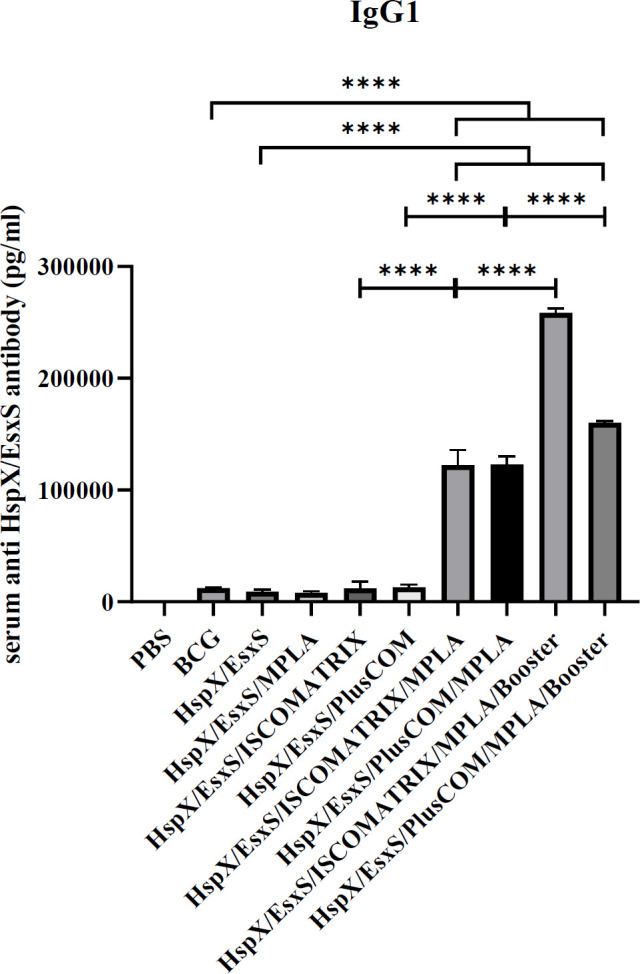
Level of IgG1 produced in the serum of mice receiving different formulations

The level of serum anti-HspX/EsxS IgG1 titers, as well as IgG2a, was significantly increased in the mice receiving HspX/EsxS/ISCOMATRIX/MPLA, HspX/EsxS/PLUSCOM/MPLA, and BCG booster formulations in comparison with HspX/EsxS and BCG vaccines (*P*<0.0001). Also, the addition of MPLA adjuvant and BCG booster formulation significantly increased the effect of HspX/EsxS/ISCOMATRIX and HspX/EsxS/PLUSCOM formulations on IgG1 antibody response (*P*<0.0001) (Figure 6).

***Assessment of sIgA antibody response***

**Figure 7:**
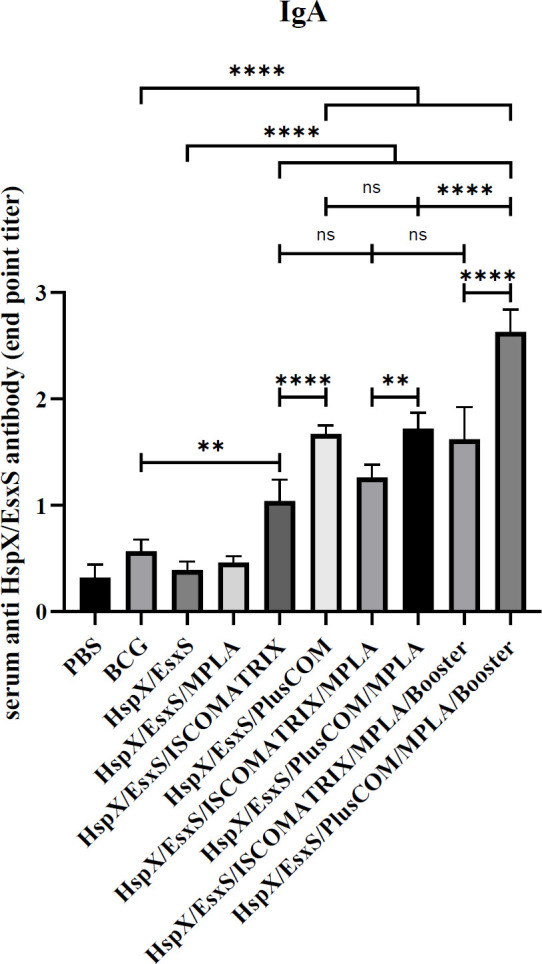
Level of anti-HspX/EsxS sIgA produced in nasal lavage of mice receiving different formulations

Anti-HspX/EsxS sIgA antibody in nasal lavage of vaccinated mice was significantly higher in HspX/EsxS/ISCOMATRIX, HspX/EsxS/PLUSCOM, HspX/EsxS/ISCOMATRIX/MPLA, HspX/EsxS/PLUSCOM/MPLA, and their BCG booster formulation in comparison with HspX/EsxS and BCG vaccines (*P*<0.0001). Furthermore, the highest level of sIgA antibody response belonged to HspX/EsxS/PlusCOM/MPLA/booster formulation. BCG booster of HspX/EsxS/PLUSCOM/MPLA significantly induced higher levels of sIgA antibody secretion than the other BCG booster formulation, ISCOMATRIX/HspX/EsxS/MPLA (*P*<0.0001). Moreover, PLUSCOM-containing formulations were able to induce higher sIgA responses than the ISCOMATRIX-containing formulation (Figure 7).

When *P*-value<0.05, differences were considered statistically significant. Significance was presented as *(*P*<0.05), **(*P*<0.01), ***(*P*<0.001), and ****(*P*<0.0001), and not significant was shown as (ns).

When *P*-value<0.05, differences were considered statistically significant. Significance was presented as *(*P*<0.05), **(*P*<0.01), ***(*P*<0.001), and ****(*P*<0.0001), and not significant was shown as (ns).

When *P*-value<0.05, differences were considered statistically significant. Significance was presented as *(*P*<0.05), **(*P*<0.01), ***(*P*<0.001), and ****(*P*<0.0001), and not significant was shown as (ns).

When *P*-value<0.05, differences were considered statistically significant. Significance was presented as *(*P*<0.05), **(*P*<0.01), ***(*P*<0.001), and ****(*P*<0.0001), and not significant was shown as (ns).

When *P*-value<0.05, differences were considered statistically significant. Significance was presented as *(*P*<0.05), **(*P*<0.01), ***(*P*<0.001), and ****(*P*<0.0001), and not significant was shown as (ns).

When *P*-value<0.05, differences were considered statistically significant. Significance was presented as *(*P*<0.05), **(*P*<0.01), ***(*P*<0.001), and ****(*P*<0.0001), and not significant was shown as (ns).

## Discussion

After 1984 when Morein and colleagues for the first time developed ISCOM-like structures, the results of several animal models and human clinical trials suggested that ISCOMATRIX-based vaccines are safe, well tolerated, and immunogenic, and able to induce strong humoral and cellular responses. The components of ISCOMATRIX adjuvant, i.e., saponin, cholesterol, and phospholipid, form a cage-like structure (40–50 nm in diameter and about -20 mV in surface charge of particle) that facilitate antigen-presentation and antigen-delivery and also show immunomodulatory properties (25, 26). The efficacy of ISCOMATRIX adjuvant is currently under evaluation for cancer and some chronic infectious diseases such as the hepatitis C virus and influenza, however, there is no study assessing ISCOMATRIX-based TB vaccines (27). 

The immunogenicity of HspX/EsxS fusion protein + ISCOMATRIX and PLUSCOM nanoadjuvants depends on the particle size and surface charge of the adjuvants. Particle size can play an important role in the activity of nanoparticles. Nanoparticles with a size less than 600 nm are also able to stimulate Th1 immune responses (20). The positive charge of the particles can also play an important role in the efficient uptake of nanoparticles by APCs and the induction of Th1 immune responses. Among the different types of *M. tuberculosis* antigens, HspX can induce strong immune responses in the early and latent phases of infection with this bacterium (16). Subcutaneous administration of ISCOMATRIX and PLUSCOM nanoadjuvants can increase the immunogenicity of HspX/EsxS fusion protein by activating Th1 cells. Therefore, positively charged PLUSCOM particles can be effective in better uptake by APCs and induction of Th1 immune responses (19). In this study, the particle size of ISCOMATRIX and PLUSCOM nanoadjuvants was about 82.4 and 180.9 nm, respectively, and the surface charges of ISCOMATRIX and PLUSCOM were -18.6 and +25.6 mv, respectively (20).

In the current study, intranasal administration of ISCOMATRIX adjuvant in combination with HspX/EsxS antigen increased immune response especially the levels of IFN-γ and IgG1, IgG2a, and sIgA antibodies compared to antigen alone. A similar result was observed with the same formulation when administrated subcutaneously (20). It shows that ISCOMATRIX can boost immunogenicity of antigen which is a main weakness of subunit antigen vaccines. Another classic ISCOMs derivative with a cage-like structure and positive surface charge is a cationic immune stimulating complex called PLUSCOM. The PLUSCOMs similar to ISCOMATRIXs can act as immunoadjuvants and can induce T cell responses against an antigen, which is the most important human body response against TB infection (23, 28, 29). Positively charged PLUSCOM nano-adjuvant in combination with TB fused antigen was able to induce higher sIgA and IFN-γ responses than negatively charged ISCOMATRIX-antigen formulation after intranasal administration. Similar results were observed in the subcutaneous route (20). One possible reason is that the positively charged PLUSCOM adjuvant strongly improves the particle-antigen uptake by the physiological surfaces such as mucosal surfaces as well as by the negatively charged immune cells particularly APCs and subsequent presentation to T cells (17, 28, 30). It is recommended that ISCOMATRIX adjuvant can be a good choice for use in the prophylactic and therapeutic vaccines. Prophylactic TB vaccine candidates are pre-exposure vaccines and similar to BCG can be administered after birth time. These types of TB vaccine candidates could be replaced with BCG or act as BCG boosters (7, 25, 31). Our results revealed that the ability of PLUSCOM/HspX/EsxS and ISCOMATRIX/HspX/EsxS formulations to elicit IFN-γ response was higher than the BCG vaccine. These vaccine formulations cannot be replaced with BCG because the results were not statistically significant in some cases. Also, addition of MPLA adjuvant into ISCOMATRIX/HspX/EsxS and PLUSCOM/HspX/EsxS formulations promoted the immune responses. The results were encouraging in intranasally vaccinated mice with formulations HspX/EsxS/ISCOMATRIX/MPLA, HspX/EsxS/PLUSCOM/MPLA, and two BCG booster groups. Similar findings were obtained for the same groups when administrated via the subcutaneous route (20). 

## Conclusion

Taken together, our study suggested that ISCOMATRIX and PLUSCOM nano-adjuvants were able to boost HspX/EsxS immunogenicity and induced higher levels of IFN-γ response and sIgA antibody secretion compared to HspX/EsxS alone, and addition of MPLA adjuvant promoted the immune responses. Furthermore, both HspX/EsxS/ISCOMATRIX/MPLA and HspX/EsxS/PLUSCOM/MPLA and their BCG booster formulation were able to induce IFN-γ response significantly higher than the BCG group. These findings demonstrate that both nanoparticles in combination with MPLA can act as immunoadjuvant. However, further *in vivo* experiments are required to confirm the efficacy of these formulations as new TB vaccines or as BCG boosters.

## Authors’ Contributions

Z M and M T conceived and designed the research. A YA and F K conducted experiments. A YA analyzed data and wrote the manuscript. All authors read and approved the final manuscript.

## Etical Statement

All applicable international, national, and institutional guidelines for the care and use of animals were followed.

## Data Availability

The datasets used and/or analyzed during the current study are available from the corresponding author upon reasonable request.

## Conflicts of Interest

The authors declare no conflicts of interest. 

## References

[1] Organization WH (2019). Global status report on alcohol and health 2018.

[2] Avarvand AY, Khademi F, Tafaghodi M, Ahmadipour Z, Moradi B, Meshkat Z (2019). The roles of latency-associated antigens in tuberculosis vaccines. Indian J Tuberc.

[3] Yousefi-Avarvand A, Tafaghodi M, Soleimanpour S, Khademi F (2018). HspX protein as a candidate vaccine against Mycobacterium tuberculosis: An overview. Front Biol.

[4] Khademi F, Yousefi-Avarvand A, Derakhshan M, Vaez H, Sadeghi R (2017). Middle east Mycobacterium tuberculosis antibiotic resistance: A systematic review and meta-analysis. Infect Epidemio Med.

[5] Ghafoor T, Ikram A, Abbassi SA, Mirza IA, Hussain A, ullah Khan I (2014). Antimicrobial sensitivity pattern of clinical isolates of Mycobacterium tuberculosis: A retrospective study from a reference laboratory in Pakistan. J Virol Microbiol.

[6] Liang J, Teng X, Yuan X, Zhang Y, Shi C, Yue T (2015). Enhanced and durable protective immune responses induced by a cocktail of recombinant BCG strains expressing antigens of multistage of Mycobacterium tuberculosis. Mol Immunol.

[7] Andersen P, Kaufmann SH (2014). Novel vaccination strategies against tuberculosis. Cold Spring Harb Perspect Med.

[8] Costa ACd, Costa-Junior AdO, Oliveira FMd, Nogueira SV, Rosa JD, Resende DP (2014). A new recombinant BCG vaccine induces specific Th17 and Th1 effector cells with higher protective efficacy against tuberculosis. PLoS One.

[9] Soleimanpour S, Farsiani H, Mosavat A, Ghazvini K, Eydgahi MRA, Sankian M (2015). APC targeting enhances immunogenicity of a novel multistage Fc-fusion tuberculosis vaccine in mice. Appl Microbiol Biotechnol.

[10] Khademi F, Derakhshan M, Yousefi-Avarvand A, Tafaghodi M, Soleimanpour S (2018). Multi-stage subunit vaccines against Mycobacterium tuberculosis: An alternative to the BCG vaccine or a BCG-prime boost?. Expert Review of Vaccines.

[11] Shirvani F, Karimi A, Rajabnejad M (2016). BCG vaccination as a prevention strategy, threats and benefits. Arch Pediatr Infect Dis.

[12] Triccas JA, Counoupas C (2016). Novel vaccination approaches to prevent tuberculosis in children. Pneumonia.

[13] McHugh KJ, Guarecuco R, Langer R, Jaklenec A (2015). Single-injection vaccines: Progress, challenges, and opportunities. J Control Release.

[14] Khademi F, Taheri RA, Avarvand AY, Vaez H, Momtazi-Borojeni AA, Soleimanpour S (2018). Are chitosan natural polymers suitable as adjuvant/delivery system for anti-tuberculosis vaccines?. Microb Pathog.

[15] Khademi F, Derakhshan M, Yousefi-Avarvand A, Tafaghodi M (2018). Potential of polymeric particles as future vaccine delivery systems/adjuvants for parenteral and non-parenteral immunization against tuberculosis: A systematic review. Iran J Basic Med Sci.

[16] Khademi F, Yousefi-Avarvand A, Derakhshan M, Meshkat Z, Tafaghodi M, Ghazvini K (2017). Mycobacterium tuberculosis HspX/EsxS fusion protein: gene cloning, protein expression, and purification in Escherichia coli. Rep Biochem Mol Biol.

[17] Khademi F, Sahebkar A, Fasihi-Ramandi M, Taheri RA (2018). Induction of strong immune response against a multicomponent antigen of Mycobacterium tuberculosis in BALB/c mice using PLGA and DOTAP adjuvant. APMIS.

[18] Khademi F, Yousefi A, Derakhshan M, Najafi A, Tafaghodi M (2019). Enhancing immunogenicity of novel multistage subunit vaccine of Mycobacterium tuberculosis using PLGA: DDA hybrid nanoparticles and MPLA: Subcutaneous administration. Iran J Basic Med Sci.

[19] Wang X, Zhang J, Liang J, Zhang Y, Teng X, Yuan X (2015). Protection against Mycobacterium tuberculosis infection offered by a new multistage subunit vaccine correlates with increased number of IFN-γ+ IL-2+ CD4+ and IFN-γ+ CD8+ T cells. PLoS One.

[20] Avarvand AY, Meshkat Z, Khademi F, Tafaghodi M (2021). Immunogenicity of HspX/EsxS fusion protein of Mycobacterium tuberculosis along with ISCOMATRIX and PLUSCOM nano-adjuvants after subcutaneous administration in animal model. Microb Pathog.

[21] McBurney WT, Lendemans DG, Myschik J, Hennessy T, Rades T, Hook S (2008). In vivo activity of cationic immune stimulating complexes (PLUSCOMs). Vaccine..

[22] Baz Morelli A, Becher D, Koernig S, Silva A, Drane D, Maraskovsky E (2012). ISCOMATRIX: A novel adjuvant for use in prophylactic and therapeutic vaccines against infectious diseases. J Med Microbiol.

[23] Lendemans DG, Myschik J, Hook S, Rades T (2005). Cationic cage-like complexes formed by DC-cholesterol, Quil-A, and phospholipid. J Pharm Sci.

[24] Khademi F, Derakhshan M, Yousefi-Avarvand A, Najafi A, Tafaghodi M (2018). A novel antigen of Mycobacterium tuberculosis and MPLA adjuvant co-entrapped into PLGA: DDA hybrid nanoparticles stimulates mucosal and systemic immunity. Microb Pathog.

[25] Drane D, Gittleson C, Boyle J, Maraskovsky E (2007). ISCOMATRIX™ adjuvant for prophylactic and therapeutic vaccines. Expert Rev Vaccines.

[26] Maraskovsky E, Schnurr M, Wilson NS, Robson NC, Boyle J, Drane D (2009). Development of prophylactic and therapeutic vaccines using the ISCOMATRIX adjuvant. Immunol Cell Biol.

[27] Bigaeva E, Doorn Ev, Liu H, Hak E (2016). Meta-analysis on randomized controlled trials of vaccines with QS-21 or ISCOMATRIX adjuvant: Safety and tolerability. PLoS One.

[28] Mehravaran A, Jaafari MR, Jalali SA, Khamesipour A, Tafaghodi M, Hojatizade M (2015). Cationic immune stimulating complexes containing soluble Leishmania antigens: Preparation, characterization and in vivo immune response evaluation. Iran J Immunol.

[29] Badiee A, Shargh VH, Khamesipour A, Jaafari MR (2013). Micro/nanoparticle adjuvants for antileishmanial vaccines: Present and future trends. Vaccine.

[30] Tafaghodi M, Khademi F, Firouzi Z (2020). Polymer-based nanoparticles as delivery systems for treatment and vaccination of tuberculosis. Nanotechnology Based Approaches for Tuberculosis Treatment.

[31] Kaufmann SH ( 2013). uberculosis vaccines: Time to think about the next generation. Semin Immunol.

